# Optimising the analysis of transcript data using high density oligonucleotide arrays and genomic DNA-based probe selection

**DOI:** 10.1186/1471-2164-8-344

**Published:** 2007-10-01

**Authors:** Neil S Graham, Martin R Broadley, John P Hammond, Philip J White, Sean T May

**Affiliations:** 1 Plant Science Division, School of Biosciences, University of Nottingham, Sutton Bonington Campus, Loughborough, LE12 5RD, UK; 2 Warwick HRI, University of Warwick, Wellesbourne, Warwick, CV35 9EF, UK; 3 The Scottish Crop Research Institute, Invergowrie, Dundee, DD2 5DA, UK

## Abstract

**Background:**

Affymetrix GeneChip arrays are widely used for transcriptomic studies in a diverse range of species. Each gene is represented on a GeneChip array by a probe-set, consisting of up to 16 probe-pairs. Signal intensities across probe-pairs within a probe-set vary in part due to different physical hybridisation characteristics of individual probes with their target labelled transcripts. We have previously developed a technique to study the transcriptomes of heterologous species based on hybridising genomic DNA (gDNA) to a GeneChip array designed for a different species, and subsequently using only those probes with good homology.

**Results:**

Here we have investigated the effects of hybridising homologous species gDNA to study the transcriptomes of species for which the arrays have been designed. Genomic DNA from *Arabidopsis thaliana *and rice (*Oryza sativa*) were hybridised to the Affymetrix Arabidopsis ATH1 and Rice Genome GeneChip arrays respectively. Probe selection based on gDNA hybridisation intensity increased the number of genes identified as significantly differentially expressed in two published studies of Arabidopsis development, and optimised the analysis of technical replicates obtained from pooled samples of RNA from rice.

**Conclusion:**

This mixed physical and bioinformatics approach can be used to optimise estimates of gene expression when using GeneChip arrays.

## Background

The use of microarrays to determine global transcriptional profiles is a valuable and widely-used tool for understanding the regulation of biological systems [1,2]. Several microarray platforms are used for these studies, including spotted arrays (using cDNAs, PCR products or oligonucleotides) and *in situ *synthesised arrays including Agilent SurePrint and Affymetrix GeneChip arrays. GeneChip arrays have a number of advantages over other arrays. For example, the uniformity and reproducibility of data from GeneChip arrays facilitates the curation of large data sets and subsequent inter-experimental comparisons [1-5]. Each gene depicted on a GeneChip array is represented by up to 16 probe-pairs, with each probe-pair consisting of a 25 base oligo perfect-match (PM) probe, designed to bind perfectly to the gene sequence, and a 25 base oligo mis-match (MM) probe, which contains a mis-match base at the 13^th ^base position, designed to measure non-specific binding [1]. This contrasts with the single cDNA or oligo probe used to assay a gene on most other arrays. However, since several signal values are generated for each gene, it is more complex to produce a single expression value for each gene, as probes within a probe-set may not have similar signal intensity due in part to the different physical hybridisation characteristics of individual probes [6]. Several normalisation algorithms are used to amalgamate probe signal values and generate a single expression value for each gene [7]. The Affymetrix system typically uses the Microarray suite (MAS) or its successor GeneChip Operating system (GCOS) to generate the gene signal values. The expression value is calculated using the "One-step Tukey's biweight algorithm", which weights the signal intensities from individual probes based on their distance from the median signal intensity of the probe-set [8]. Other normalisation algorithms have been developed that use the signal intensities from all the arrays in an experiment to determine gene expression values. These include "Model-Based Expression Indexes" [9,10] and the "Robust Multiarray Average" (RMA) algorithms [11]. With these methods, the probe response pattern across all genes is fitted across all the arrays used in an experiment and a robust estimate of the background signal is modelled and the data adjusted accordingly. These models have been developed further to account for the physical binding properties of the probes. Examples of these models are "Positional-Dependent-Nearest-Neighbour model" [12] and GC-RMA [13].

The design of Affymetrix GeneChip arrays also enables the transcriptional profiling of species for which the arrays were not designed [14-23]. For example, Hammond *et al*. [22,23] used a mixed physical and bioinformatic method, which involved hybridising genomic DNA (gDNA) from a species onto a GeneChip array of a heterologous species. A parser script, written in Perl, was developed to generate probe-masking files by removing probe-pairs whose PM probe signal intensity value was below a user-defined gDNA hybridisation intensity threshold. These probe-masking files, containing the retained probe-pairs, were then used for analysis of transcriptional data. Using this technique increased the sensitivity of using an *Arabidopsis *ATH1 array to study transcriptional responses of *Brassica oleracea *to phosphorus stress [22]. This technique also allowed the shoot transcriptional profiles of two closely related Brassicaceae species, *Thlaspi caerulescens *and *T. arvense *to be compared more satisfactorily [23]. The technique has also been used with human chips to analyse several heterologous animal species such as horse, sheep and guinea pig (data not shown).

The aim of this study was to determine if gDNA-based probe selection can improve estimates of gene expression in homologous species transcriptome analyses. We hybridised gDNA from *Arabidopsis thaliana *and rice (*Oryza sativa*) to the Affymetrix Arabidopsis ATH1 and Rice Genome GeneChip arrays respectively. Only those probe-pairs whose PM probe hybridised to gDNA above defined signal intensity thresholds were retained and these were used to reanalyse previously published transcriptome data sets. Two published studies of Arabidopsis shoot development from the AtGenExpress project [24], and six technical replicates of pooled rice RNA spiked with two different concentrations of bacterial control genes (PlexDB, accession number OS1 [25,26]) were reanalysed using this approach. Probe selection based on gDNA hybridisation was also compared to the random removal of probe-pairs. Probe selection increased the number, and altered the identity of genes identified as significantly differentially expressed in the Arabidopsis study and optimised the analysis of pooled rice RNA.

This mixed physical and bioinformatics approach can be applied post-experiment and is applicable to all species for which Affymetrix GeneChip arrays have been developed including human chips.

## Results and discussion

### Genomic DNA hybridisations and probe selection

The aim of the study was to investigate the effects of using a mixed physical and bioinformatics probe-masking approach on the study of the transcriptomes of two species. *Arabidopsis thaliana *and rice gDNA was hybridised to the Affymetrix Arabidopsis ATH1 and Rice Genome GeneChip arrays respectively. A probe-pair was selected if its perfect-match (PM) gDNA hybridisation signal intensity was greater than a series of defined thresholds (ranging from 0 [no probe selection] to 1000), using a .cel file parser script written in Perl [22]. The probe-pairs retained in the .cdf files had good homology to the gDNA as defined by their gDNA signal intensities, and were used to analyse published transcriptome data at the defined thresholds.

**Figure 1:**
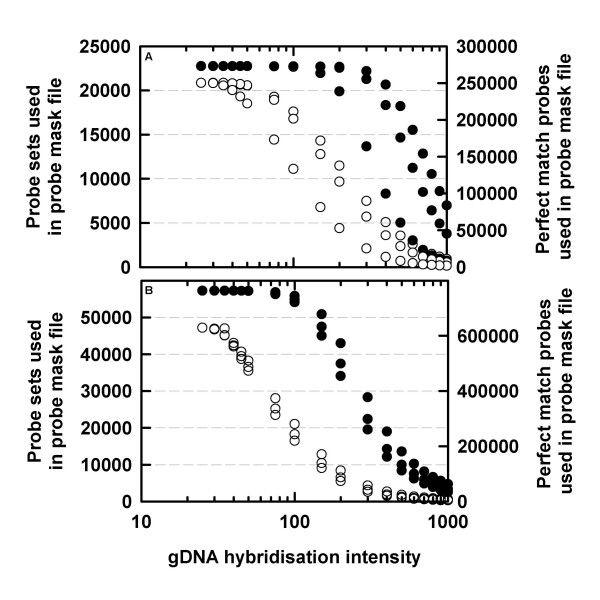
Number of (A) *Arabidopsis thaliana *and (B) *Oryza sativa *probe-pairs and probe-sets used to study the transcriptome of *A. thaliana *and *O. sativa *respectively, as a function of the gDNA hybridisation intensity thresholds used to generate the probe-mask files. Filled circles are scaled to the left-hand *y*-axis (i.e. probe-sets retained within probe-mask files) and unfilled circles are scaled to the right-hand *y*-axis (i.e. probe-pairs retained within probe-mask files). Three gDNA hybridisations were performed for each species.

The Arabidopsis ATH1 GeneChip array contains 22,746 probe-sets, representing approximately 24,000 genes. As expected, Arabidopsis gDNA hybridised well to the ATH1 GeneChip array. The retention of probe-pairs in the probe-mask files declined at higher gDNA hybridisation intensity thresholds (Figure 1A). As expected, the number of probe-sets retained in the probe-mask files declined at a slower rate than the number of probe-pairs retained at higher gDNA hybridisation intensity thresholds (Figure 1A). These results are consistent with those obtained by hybridising gDNA from *B. oleracea*,*T. caerulescens *or *T. arvense *to the Arabidopsis ATH1 array [22,23]. The retention of probe-pairs at higher gDNA hybridisation intensity thresholds was lower in one of the three Arabidopsis replicates. This may be due to technical rather than biological variation since gDNA should be consistent when isolated from the same plant genotype. Notably, the three Arabidopsis replicates had a Spearman's similarity measure of 1, as calculated by the condition tree function in GeneSpring GX (Agilent Technologies, Palo Alto, CA, USA; data not shown). Whilst it may be feasible to use model-based normalisation strategies to adjust for probe-level differences in gDNA hybridisation intensities between biological replicates, we have adopted a more conservative probe-masking strategy. This strategy was based on retaining only those probe-pairs whose PM gDNA hybridisation intensity was sufficiently high in all three biological replicates.

The Affymetrix Rice Genome array is designed to analyse 48,564 *Oryza sativa cv. japonica *and 1,260 *O. sativa cv. indica *transcripts [26]. Genomic DNA was extracted from one *japonica *(Sharbati) and two *indica *varieties (385 and Super). As with Arabidopsis, the rice gDNA hybridised well to the array. The retention of probe-pairs and probe-sets decline at higher gDNA hybridisation intensity thresholds (Figure 1B). The three replicate rice gDNA hybridisations produced similar results across the range of gDNA hybridisation intensity thresholds.

These data show that in both Arabidopsis and rice, hybridisation efficiencies between individual PM probes and their target transcript vary within and between probe-sets. Variation in hybridisation efficiencies could be due to the physical binding properties of probes and the number of targets within the genome. For example, hybridisation efficiency is reduced when probes and their targets form secondary structures, when probes have unresponsive binding affinities, when interactions with fluorescent labels are unfavourable, and when non-specific binding occurs [6,27].

### Analysis of data sets from the AtGenExpress project

The AtGenExpress project has produced a large quantity of high-quality gene expression data for the model plant Arabidopsis [24]. It includes GeneChip array data from developmental time-course experiments and experiments in which plants were subjected to hormones, abiotic or biotic stresses. Two shoot developmental time-course experiments from the AtGenExpress project were reanalysed here using a gDNA based probe selection: Data Set A, in which different aged rosette leaves (number 2, 4, 6, 8, 10 and 12) were taken from 17 day old plants, and Data Set B, in which pooled rosette leaves were taken from 7, 14 and 21 day old plants. All conditions comprised three biological replicates. Data Sets A and B were filtered for genes that were differentially expressed between one or more conditions within each experiment using probe-mask files generated at different gDNA hybridisation intensity thresholds.

**Figure 2:**
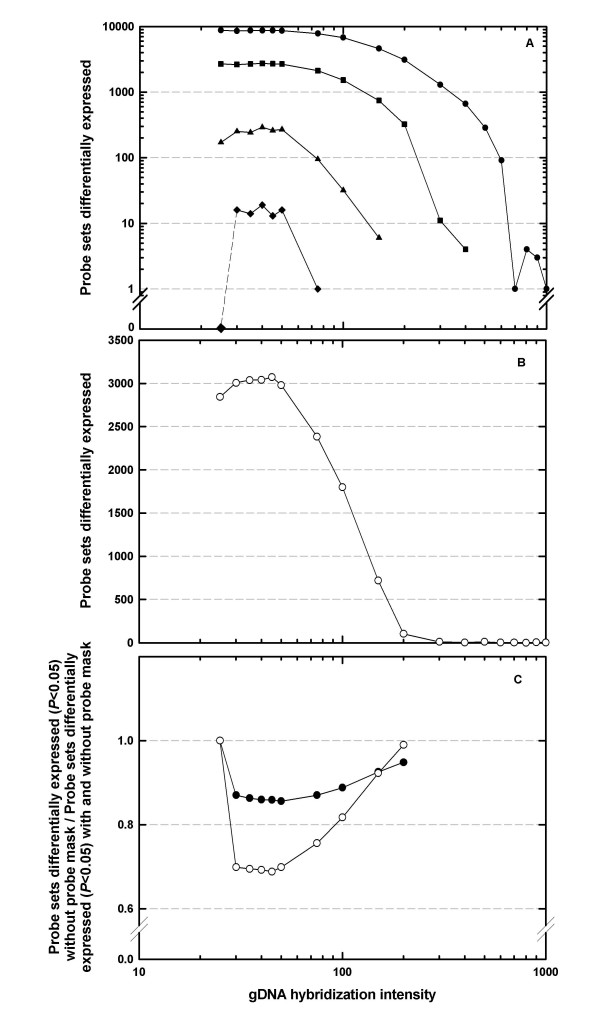
(A, B) Genes identified as significantly differentially expressed (*p *< 0.05) in *Arabidopsis thaliana*, as a function of the gDNA hybridisation intensity threshold used to generate probe-mask files, for two AtGenExpress experiments [24]. (A) RNA was extracted from leaves 2, 4, 6, 8, 10 and 12, sampled from 17 d old plants (n = 3), labelled and hybridised to the *A. thaliana *ATH1-121501 GeneChip array, and data were normalised to the median expression value of each gene across all leaves. The number of genes identified as differentially expressed in one, two, three and four of the six conditions are represented by filled circles, squares, triangles and diamonds respectively. (B) RNA was extracted from whole rosettes of 7, 14, and 21 d old plants (n = 3), labelled and hybridised to the *A. thaliana *ATH1-121501 GeneChip array, and normalised to the median expression value of each gene across all time points. (C) Genes identified as significantly differentially expressed (*p *< 0.05) in the absence of a probe-mask, as a proportion of the sum of all genes significantly differentially expressed (*p *< 0.05) when analyses were conducted with and without a corresponding probe-mask. Filled circles represent Data Set A, open circles represent Data Set B.

For Data Set A, the number of genes identified as significantly differentially expressed increased slightly, and then decreased, as a function of the gDNA hybridisation intensity threshold used in the probe-mask file (Figure 2A). This observation is consistent with transcriptome analysis of other Brassicaceae species using the ATH1 GeneChip array, when probe-mask files based on the hybridisation of heterologous species gDNA to the ATH1 GeneChip array were used [22,23]. A similar pattern was observed when Data Set B was analysed (Figure 2B).

In addition to affecting the number of genes identified as significantly differentially expressed, gDNA-based probe-masks also affected the identity of genes significantly differentially expressed between treatment conditions in the Arabidopsis Data Sets A and B (Figure 2C). Thus, the number of genes identified as differentially expressed (*p *< 0.05) in the absence of a probe-mask were expressed as a proportion of the sum of all genes differentially expressed (*p *< 0.05) both with and without probe-masks. At low gDNA hybridisation intensity thresholds, and for both Data Sets A and B, genes significantly (*p *< 0.05) differentially expressed in the absence of a probe-mask declined markedly as a proportion of the sum of all genes significantly differentially expressed (*p *< 0.05) both with and without probe-masks, before returning to unity at gDNA hybridisation intensity thresholds >200 (Figure 2C). This decline in the proportion of genes represented in the analysis of data without probe-masking corresponds to slight increases in the total number of genes differentially expressed when probe-masking was used (Figure 2A, B). Therefore, gDNA based probe selection affects both the number and the identity of genes which are identified as significantly differentially expressed in these two Arabidopsis experiments.

The effects of gDNA-based probe removal on estimates of gene expression differences was compared to the effects of random removal of probe-pairs using Arabidopsis Data Set B. Software to simulate random probe-pair removal (Xspecies Version 2.0) has been developed and is freely available [31]. Random probe-pair removal of 1, 2, 5, 10, 20, 50, 75 and 90% of probe-pairs was repeated three times on one of the Arabidopsis gDNA .cel files. Thus, the random removal of, for example, 50% of the probe-pairs (i.e. 125,103 probe-pairs) from Arabidopsis ATH1 GeneChip should remove an average of 11 probe-sets (i.e. 0.5^11 ^* 22,746 = 11.1). Here, in three random simulations of 50% probe-pair removal, 127,583, 127,951 and 127,882 probe-pairs were removed (x¯
 MathType@MTEF@5@5@+=feaafiart1ev1aaatCvAUfKttLearuWrP9MDH5MBPbIqV92AaeXatLxBI9gBaebbnrfifHhDYfgasaacH8akY=wiFfYdH8Gipec8Eeeu0xXdbba9frFj0=OqFfea0dXdd9vqai=hGuQ8kuc9pgc9s8qqaq=dirpe0xb9q8qiLsFr0=vr0=vr0dc8meaabaqaciaacaGaaeqabaqabeGadaaakeaacuWG4baEgaqeaaaa@2E3D@ = 127,805 ± 113 SEM) and the corresponding probe-set removal was 12, 14 and 15.

**Figure 3:**
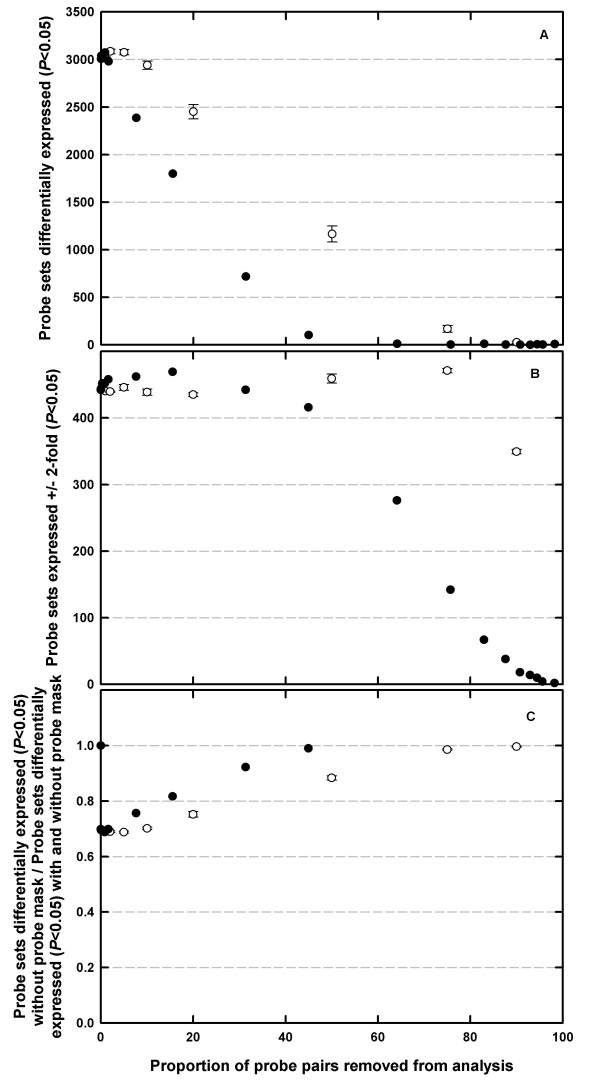
Genes (A) differentially (*p *< 0.05) or (B) ± 2-fold differentially (*p *< 0.05) expressed in one or more of the three treatment conditions in *Arabidopsis thaliana *in which RNA was extracted from whole rosettes of 7, 14, and 21 d old plants, labelled and hybridised to the *A. thaliana *ATH1-121501 GeneChip array ([24]; n = 3) and normalised to the median expression value of each gene across all time points. Filled circles represent probe-pairs removed from the analysis using gDNA hybridisation intensity thresholds. Open circles represent random removal of 1, 2, 5, 10, 20, 50, 75 and 90% of probe-pairs from an Arabidopsis gDNA .cel files. Random probe removal was repeated on three occasions (error bars represent ± S.E.M). (C) Genes identified as significantly differentially expressed (*p *< 0.05) in the absence of a probe-mask, as a proportion of the sum of all genes significantly differentially expressed (*p *< 0.05) when analyses were conducted with and without a corresponding probe-mask. All panels are expressed as a function of the percentage of probe-pairs removed from the analysis either by random simulation, or by gDNA hybridisation.

Unsurprisingly, random probe-pair removal also affected probe-set expression estimates. Random probe-pair removal increased the number of genes identified as significantly differentially expressed (P < 0.05) compared to corresponding gDNA-based probe-masks, especially when a large proportion of the probe-pairs were removed from the analysis (Figure 3A). However, based on the assumption that gDNA hybridisation will tend to remove less-informative probe-pairs compared to a random probe selection strategy, due to differences in probe-level hybridisation efficiencies, we can hypothesise that gDNA based probe-masking is a more appropriate analysis step. Consistent with this hypothesis, random probe-removal of <40% of all probe-pairs decreased the number of genes identified as significantly differentially expressed (P < 0.05) by > ± 2-fold between the 7 and 14-d old rosette leaves in Data Set B compared to corresponding gDNA-based probe-masks (Figure 3B). Furthermore, probe-sets identified as significantly differentially expressed using a gDNA-based probe-mask were more similar to the identity of probe-sets significantly differentially expressed in the absence of a probe-mask, than were those probe-sets identified using a corresponding random probe-removal mask (Figure 3C). The hypothesis that targeted gDNA-based probe-masking is an appropriate analysis procedure should now be tested for further ecotypes and for other species using the software developed in this study [31].

An alternative to gDNA based probe selection is to filter out probes based on poor RNA hybridisation intensities [28]. Thus, when data from the human HG-U133A GeneChip array was analysed using a probe-mask file based on RNA hybridisation intensity, the number of probe-sets called 'present' by the MAS 5.0 algorithm increased 1.5-fold [6]. However, in contrast to gDNA-based probe selection strategies, selection of probes based on the RNA hybridisation signal will bias the anlaysis towards those transcripts which are most abundant in the sample used.

### Analysis of *Arabidopsis thaliana *reference genes from AtGenExpress project

**Figure 4:**
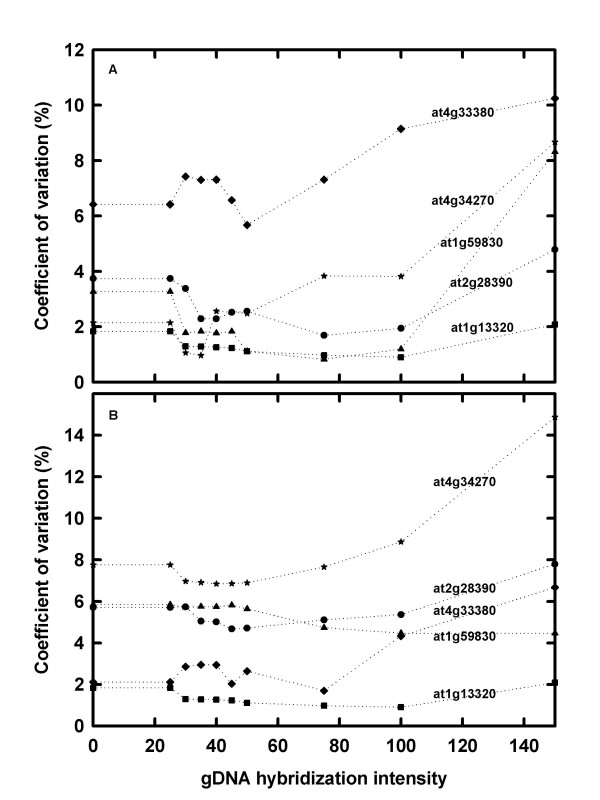
The percentage coefficient of variation (% CV) for five *Arabidopsis thaliana *reference genes, from two AtGenExpress experiments [24]. (A) RNA was extracted from leaves 2, 4, 6, 8, 10 and 12, sampled from 17 d old plants (n = 3), labelled and hybridised to the *A. thaliana *ATH1-121501 GeneChip array, and data were normalised to the median expression value of each gene across all leaves. (B) RNA was extracted from whole rosettes of 7, 14, and 21 d old plants (n = 3), labelled and hybridised to the *A. thaliana *ATH1-121501 GeneChip array, and normalised to the median expression value of each gene across all time points.

A set of references genes whose expression varied little between tissue types and during development has been reported for the AtGenExpress data [29]. These genes were identified by calculating the percentage coefficient of variation (% CV) of all genes across all samples; genes with the lowest % CV and with low expression values were selected and confirmed by real-time PCR [29]. The % CV of five of these reference genes (At4g3380, At4g34270, At1g59830, At2g 28390 and At1g13320), whose %CV were the lowest across the AtGenExpress developmental series [29], were calculated across the range of gDNA hybridisation intensity thresholds for Data Sets A and B. In general, the % CV was lowest when a gDNA hybridisation intensity threshold of 30 to 50 was used (Figure 4A, B). The expression values for the five genes also varied when the gDNA probe-selection method was applied. For example, with Data Set A (leaf number 2), At1g13220 expression value decreases from 404.4 (no probe selection) to 356.1 (threshold of 40) before increasing again to 535.4 (at a threshold of 200). The expression values of this gene in all the leaf samples of this data set follow the same pattern, with the value first decreasing then increasing. In contrast a different pattern is seen with Data Set B. The expression value (7 d old rosette leaves) increases as the gDNA hybridisation intensity threshold increases, from 502.9 (no probe selection) to 1015 (at a gDNA hybridisation intensity threshold of 200). These results demonstrate that the probe-selection method can have a profound effect on the expression value obtained for individual genes and illustrates how the identity of genes identified as significantly differentially expressed will differ when a probe-selection method is applied.

### Analysis of a rice data set

**Figure 5:**
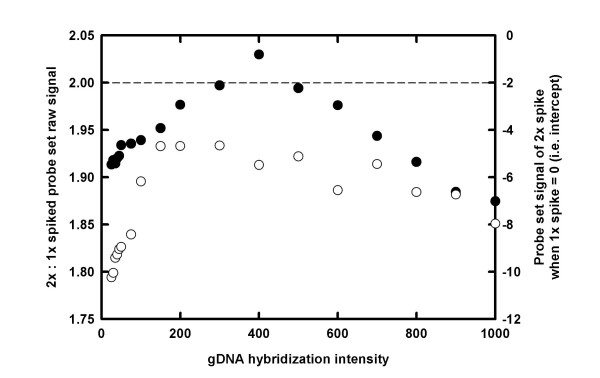
The relative expression of bacterial control transcripts (n = 27) in *Oryza sativa *RNA samples as a function of the genomic DNA (gDNA) hybridisation intensity thresholds used to make a probe-mask file. RNA was extracted from *O. sativa*, labelled and hybridised to rice genome GeneChip arrays. Samples were spiked with bacterial control transcripts at either 1.8 pM or 3.6 pM (n = 3). Data were obtained from the PLEXdb database ([25, 30]; accession number OS1, submitted by T Close). Data were normalised to the median expression value of each gene across all six samples. Filled circles are scaled to the left-hand *y*-axis, and represent the ratio of bacterial control transcript mean signals in samples spiked at 3.6 pM and 1.8 pM, i.e. this number defines the slope of a linear regression with an expected value of 2. Unfilled circles are scaled to the right-hand *y*-axis and represent the bacterial control transcript mean signals in samples spiked at 3.6 pM, when the corresponding mean signal in samples spiked at 1.8 pM equals zero, i.e. this number defines the *y*-intercept term of a linear regression with an expected value of 0.

To further investigate whether transcriptome analysis can be optimised by selecting probe-pairs on a GeneChip array that hybridise well to gDNA from a homologous species, a Rice Genome GeneChip array data set was reanalysed. The data set was obtained from the PLEXdb database (accession number OS1, submitted by T Close) [25,26,30]. This data set consists of six hybridisations of the same pooled RNA sample, with three of the samples spiked with bacterial control transcripts at a concentration of 1.8 pM and three samples spiked with bacterial control transcripts at a concentration of 3.6 pM. In total, four bacterial transcripts, represented by nine probe-sets, were present in the spike mixture. Since there are three generations of the probe-sets (designed to different criteria) present on the array, a total of 27 probe-sets were used in the analysis. The data was analysed by calculating the ratio of the bacterial control genes between GeneChip arrays spiked with 1.8 pM bacterial control transcripts and GeneChip arrays spiked with 3.6 pM bacterial control transcripts at a range of gDNA hybridisation intensity thresholds. As the gDNA hybridisation intensity threshold increased, the ratio of the control genes from the two pools of differentially-spiked samples increased to the expected value of 2 at a gDNA hybridisation threshold of 300, before declining again as the gDNA hybridisation intensity threshold was increased still further (Figure 5). Similarly, the intercept of the linear regression increased towards the expected value of 0, before decreasing again as the gDNA hybridisation intensity threshold increased (Figure 5).

## Conclusion

The results presented here demonstrate that a probe-selection method can be used to optimise transcriptome analyses. Genomic DNA from a homologous species can be hybridised to its respective GeneChip array, and a subset of probe-pairs can be selected based on the hybridisation efficiency between the PM probe and its target sequence. This subset of probe-pairs can then be used in the subsequent transcriptome analysis. The change in apparent expression levels can lead to differences in the number and identity of genes identified as significantly differentially expressed between experimental conditions. The method can alter the apparent expression level of individual genes although the effect is not consistent across all genes. The approach can be applied post-experiment and is applicable to all species for which Affymetrix GeneChip arrays have been developed.

## Methods

### Genomic DNA extractions

Three replicate samples of gDNA were extracted from *Arabidopsis thaliana *(Columbia-0, Nottingham Arabidopsis Stock Centre, N1902)leaf tissue using a Qiagen DNeasy plant mini kit according to the manufacturer's instructions (Qiagen Ltd., Crawley, UK). Rice grains from three varieties (Basmati 385, Basmati Super, Sharbati) were ground in liquid nitrogen to a fine powder using a pestle and mortar and 100 mg of ground tissue was transferred to a 2.0 ml eppendorf tube. To this, 750 μl extraction buffer (100 mM Tris pH 8.0, 50 mM EDTA pH 8.0, 0.5 M NaCl, 10 mM β-mercaptoethanol) and 50 μl 10% SDS were added. Following incubation at 70°C for 10 min, 250 μl of 5 M potassium acetate was added and the sample incubated on ice for 20 min. The sample was then centrifuged at 11,600 *g *for 15 min; the supernatant was removed and added to a 2.0 ml eppendorf tube containing 500 μl isopropanol and incubated at -20°C for 20 min. The sample was centrifuged at 11,600 *g *for 15 min to pellet the DNA. The supernatant was removed and the DNA pellet washed with 70% ethanol. After washing, the pellet was air dried for 30 min then dissolved in 50 μl ultra-pure water.

### Genomic DNA hybridisations and probe selection

All six samples of gDNA (500 ng) were labelled using the Bioprime DNA labelling system according to the manufacturer's instructions (Invitrogen, Paisley, UK) and hybridised to the Affymetrix Arabidopsis ATH1-121501 or Rice Genome GeneChip arrays for 16 h at 45°C using standard Affymetrix hybridisation protocols (Affymetrix, Santa Clara, CA, USA). The GeneChip arrays were scanned using an Affymetrix 3000 GeneArray scanner and gDNA cell intensity files (.cel files) were generated using the Microarray Analysis Suite (MAS Version 5, Affymetrix). Probe-pairs from the gDNA .cel files were selected using a .cel file parser script [31] which produces a probe-mask file (.cdf) compatible with a range of microarray analysis packages and containing only probe-pairs in which the perfect-match probe has a gDNA hybridisation intensity greater than the user defined gDNA hybridisation threshold [22]. The probe-mask files were produced using the following gDNA hybridisation intensity thresholds: 25, 30, 35, 40, 45, 50, 75, 100, 150, 200, 300... 1000. A probe-set was removed from the analysis once the gDNA hybridisation intensity for all 11 of its probe-pairs fell below the designated threshold.

### Re-analysis of transcriptome data

At each gDNA hybridisation intensity threshold a single .cdf probe-mask file was created for both Arabidopsis and rice, based on the three replicate gDNA hybridisations. This was achieved by an iterative process using the .cel file parser script. Initially, the script was run with using the gDNA .cel file from replicate one and the ATH1-121501 or Rice Genome .cdf file [32]. This generated a new .cdf file, 'Rep1.cdf', containing probe-pairs in which the perfect-match probe had a gDNA hybridisation intensity greater than the user defined gDNA hybridisation threshold, based on replicate one. This process was repeated using the gDNA .cel file from replicate two and the 'Rep1.cdf'. This generated a second .cdf file, 'Rep12.cdf', containing probe-pairs in which the perfect-match probe had a gDNA hybridisation intensity greater than the user defined gDNA hybridisation threshold, based on replicates one and two. Finally, the process was repeated using the gDNA cel file from replicate three and the 'Rep12.cdf'. This generated the final .cdf file, 'Rep123.cdf', containing probe-pairs in which the perfect-match probe had a gDNA hybridisation intensity greater than the user defined gDNA hybridisation threshold, based on replicates one, two and three. This .cdf file was used for analysing the transcriptional data sets.

The RNA .cel files for the Arabidopsis datasets were obtained from the AtGenExpress leaf development series of experiments [29] curated at NASCarrays [33] (Experiment Reference Number: NASCARRAYS-150). Data Set A consisted of samples from different aged rosette leaves (numbered 2, 4, 6, 8, 10 and 12) taken from 17 d old plants and Data Set B consisted of samples of rosette leaves taken from 7, 14 and 21 d old seedlings. All conditions had three replicate samples. Full descriptions of these samples are available from NASCarrays [33]. The rice RNA .cel files were obtained from PLEXdb database (accession number OS1) [25]. This data set consists of six technical replicates based on hybridisations of the same pooled RNA sample. Three of these samples were spiked with bacterial control transcripts at a concentration of 1.8 pM and three samples were spiked with bacterial control transcripts at a concentration of 3.6 pM. The probe-sets used in the analysis were: AFFX-BioB-3_at, AFFX-BioB-5_at, AFFX-BioB-M_at, AFFX-BioC-3_at, AFFX-BioC-5_at, AFFX-BioDn-3_at, AFFX-BioDn-5_at, AFFX-CreX-3_at, AFFX-CreX-5_at, AFFX-r2-Ec-bioB-3_at, AFFX-r2-Ec-bioB-5_at, AFFX-r2-Ec-bioB-M_at, AFFX-r2-Ec-bioC-3_at, AFFX-r2-Ec-bioC-5_at, AFFX-r2-Ec-bioD-3_at, AFFX-r2-Ec-bioD-5_at, AFFX-r2-P1-cre-3_at, AFFX-r2-P1-cre-5_at, AFFX-Os-r2-Ec-bioB-3_at, AFFX-Os-r2-Ec-bioB-5_at, AFFX-Os-r2-Ec-bioB-M_at, AFFX-Os-r2-Ec-bioC-3_at, AFFX-Os-r2-Ec-bioC-5_at, AFFX-Os-r2-Ec-bioD-3_at, AFFX-Os-r2-Ec-bioD-5_at, AFFX-Os-r2-P1-cre-3_s_at, AFFX-Os-r2-P1-cre-5_s_at

Initially, the RNA .cel files were loaded into GeneSpring GX (Agilent Technologies, Palo Alto, CA, USA) using the RMA normalisation algorithm [11]. The ATH1-121501 or Rice Genome .cdf files (obtained from Affymetrix [32]), representing analysis without gDNA based probe selection files, was then used to normalise the RNA .cel files. These RNA .cel files were then reanalysed using the gDNA .cdf files ('Rep123.cdf') generated at a range of gDNA hybridisations thresholds from 25 to 1000 (see above). This generated 18 data sets within each experiment. Within each data set a further normalisation was performed to standardise the expression data to the median expression value for each probe-set across all replicates (i.e. n = 3, as defined by the original experimenters). Within each data set, genes whose expression differed significantly between one or more condition (*p *< 0.05) were identified using a Welch's t-test and the Benjamini-Hochberg False Discovery Rate (FDR) multiple testing correction. For Arabidopsis Data Set A, data were filtered to identify genes whose expression differed significantly in at least 1, 2, 3 or 4 of the 6 conditions.

## Abbreviations

GCOS – GeneChip Operating system

gDNA – Genomic DNA

MAS – Microarray suite

MM – Mis-match probe

PM – Perfect match probe

## Authors' contributions

NSG and MRB contributed to the conception and design of the study, to data analysis, and to the drafting and editing of the manuscript. PJW and JPH contributed to the conception and design of the study, and to the drafting and editing of the manuscript. STM contributed to the conception and design of the study, to the drafting and editing of the manuscript and developed the perl scripts. All authors read and approved the final manuscript.
